# Physiochemical characteristics and fermentation ability of milk from Czech Fleckvieh cows are related to genetic polymorphisms of *β*-casein, *κ*-casein, and *β*-lactoglobulin

**DOI:** 10.5713/ajas.17.0924

**Published:** 2018-12-21

**Authors:** Jitka Kyselová, Kateřina Ječmínková, Jitka Matějíčková, Oto Hanuš, Tomáš Kott, Miloslava Štípková, Michaela Krejčová

**Affiliations:** 1 Institute of Animal Science, Department of Genetics and Animal Breeding, 104 00 Prague, Czech Republic; 2 Research and Breeding Institute of Pomology Holovousy, 508 01 Hořice, Czech Republic; 3 Dairy Research Institute, 160 00 Prague 6, Czech Republic

**Keywords:** Acid Fermentation, Cattle, Genotype, Milk Acidity, Milk Protein

## Abstract

**Objective:**

The aim of the study was to find a possible association between the *β*- and *κ*-casein and *β*-lactoglobulin genotypes and important milk physiochemical and technological characteristics such as acidity, alcohol stability, the contents of some minerals and the parameters of acid fermentation ability (FEA) in Czech Fleckvieh Cattle.

**Methods:**

Milk and blood samples were collected from 338 primiparous Czech Fleckvieh cows at the same stage of lactation. The genotypes of individual cows for *κ*-casein (alleles *A*, *B*, and *E*) and *β*-lactoglobulin (alleles *A* and *B*) were ascertained by polymerase chain reaction-restriction fragment length polymorphism, while their *β*-casein (alleles *A*^1^, *A*^2^, *A*^3^, and *B*) genotype was determined using melting curve genotyping analysis. The data collected were i) milk traits including active acidity (pH), titratable acidity (TA), alcohol stability (AS); calcium (Ca), phosphorus (P), sodium (Na), magnesium (Mg), and potassium (K) contents; and ii) yoghurt traits including active acidity (Y-pH), titratable acidity (Y-TA), and the counts of both *Lactobacilli* and *Streptococci* in 1 mL of yoghurt. A linear model was assumed with fixed effects of herd, year, and season of calving, an effect of the age of the cow at first calving and effects of the casein and lactoglobulin genotypes of *β*-CN (*β*-casein, *CSN2*), *κ*-CN (*κ*-casein, *CSN3*), and *β*-LG (*β*-lactoglobulin, *LGB*), or the three-way interaction between those genes.

**Results:**

The genetic polymorphisms were related to the milk TA, AS, content of P and Ca, Y-pH and *Lactobacilli* number in the fresh yoghurt. The *CSN3* genotype was significantly associated with milk AS (p<0.05). The effect of the composite *CSN2-CSN3-LGB* genotype on the investigated traits mostly reflected the effects of the individual genes. It significantly influenced TA (p<0.01), Y-pH (p<0.05) and the log of the *Lactobacilli* count (p<0.05).

**Conclusion:**

Our findings indicate that the yoghurt fermentation test together with milk proteins genotyping could contribute to milk quality control and highlight new perspectives in dairy cattle selection.

## INTRODUCTION

The genetic polymorphism of milk proteins (MP) has been studied since the second half of the last century. Further research was accelerated when the relationship between economically important milk performance traits and single nucleotide polymorphisms (SNPs) in coding and regulatory regions of MP candidate genes was revealed. Consequently, the effects of SNPs on the quality of dairy products and human nutrition are considered for applications in animal breeding.

In cattle, characterization of the amino acid composition and genetic variants of the six main MP (*α_S1_*-casein, *α_S2_*-casein, *β*-casein [*β*-CN, *CSN2*], *κ*-casein [*κ*-CN, *CSN3*], *β*-lactoglobulin [*β*-LG, *LGB*], and *α*-lactalbumin) was thoroughly reviewed by Farrel et al [1]. Numerous scientific reports supported the relevance of *β*-, *κ*-casein, and *β*-lactoglobulin genetic polymorphisms to milk yield and milk composition traits [2,3]. Several authors described joint effects of *CSN2*, *CSN3*, and *LGB* genotypes, and it is generally considered more appropriate to analyse haplotypes or composite genotypes rather than single genes in dairy cattle selection [4,5].

The physiochemical composition and other technological parameters of milk are very important in terms of proper milk processability, product quality and dairy profitability. There is overall agreement about the relevance of casein genetic diversity to MP coagulation properties and cheese quality and yield. Nevertheless, the studies about milk acidity, alcohol stability (AS), mineral content and acid fermentation in relation to MP polymorphisms have been rather limited. Comin et al [6] and Cecchinato et al [7] investigated the effects of *CSN2* and *CSN3* genotypes on milk pH and titratable acidity. Other works noted the relevance of the *LGB* polymorphism to milk pH [8] and mineral content [9]. The study of Hallen et al [10] aimed to analyse the effect of *β*-LG and the two most polymorphic *β*-CN and *κ*-CN genotypes on acid-induced gelation of bovine milk. Authors only described a significant overall effect of *β*-LG genotype on rheological properties of the glucono-δ-lactone induced milk gels. Their findings suggested that MP genetic polymorphism can be important for production of fermented milk products. Penasa et al [11] quantified the effect of composite *β*- and *κ*-casein genotypes on the genetic variation in milk pH and titratable acidity (TA). The inclusion of casein genotypes in tested animal model led to a decrease of 18, and 23% in the genetic variance for pH, and TA, respectively. This study reported most of all the notable influence of composite *CSN2* and *CSN3* genotypes on milk coagulation properties defined as rennet coagulation time and curd firmness. To the best of our knowledge, the association between casein and lactoglobulin genotypes and both the ability for acid fermentation with yoghurt beneficial culture and yoghurt physiochemical traits has not been estimated yet.

The aim of the study was to find a possible association between the *CSN2*, *CSN3*, and *LGB* genotypes and important technological milk characteristics such as pH, titratable acidity, AS, certain mineral contents and the parameters of acid fermentation ability in Czech Fleckvieh Cattle.

## MATERIALS AND METHODS

### Cows and sampling

All experiments were performed in accordance with relevant guidelines and regulations recommended by the Ministry of Agriculture of the Czech Republic. The animal blood samples were collected by an experienced veterinarian, and rules of the law approving ethical handling of animals were upheld.

The study was carried out in cows from four commercial farms. A total of 338 healthy Czech Fleckvieh cows in their first lactation were included in the investigation. Czech Fleckvieh Cattle (CFC) is a Simmental-type, dual-purpose (milk and meat) original breed widely used in the Czech Republic. All cows reached complete (305 days) lactation. Their average recorded milk yield was 7,167 kg of milk with 3.86% fat, 3.33% protein, and 4.88% lactose content. They were fed total mixed ration consisting of corn and Lucerne silage and enriched grain concentrate. The milk samples for laboratory analysis were collected during the same stage of individual lactation, after the first 60 days of milking. They were preserved with 0.04% Bronopol and analysed after cooled transport (<10°C) to the laboratory. Blood samples were collected aseptically from *v. jugularis* in tubes containing anticoagulant (0.5 mM ethylenediaminetetraacetic acid).

### Genotyping of milk protein variants

Genomic DNA was extracted from whole blood using a standard protocol with an ABI PRISM Nucleic Acid PrepStation 6100 instrument (Applied Biosystems Co, Foster City, CA, USA). Alleles *A*, *B*, and *E* of *CSN3* and *A* and *B* of *LGB* were typed by polymerase chain reaction-restriction fragment length polymorphism (PCR-RFLP), as described previously [12]. The gene polymorphisms (SNP) were analysed on the basis of nucleotide exchanges in codons 148 and 155 of exon 4 in the *CSN3* and in codons 64 and 118 of exon 4 in the *LGB*. The restriction fragment length polymorphism-PCR products were visualized by electrophoresis on 2% agarose gel and documented using GeneSys software of G:Box F3 fluorescence system (Syngene, Cambridge, UK). Exon 7, which harbours the alleles *A*^1^, *A*^2^, *A*^3^, and *B* of *CSN2* was analysed with melting curve genotyping in the real-time PCR Light Cycler 480 instrument (Roche Diagnostics, Mannheim, Germany). The principle behind this method was described in detail by Sztankóová et al [13]. A 498-bp fragment containing exon 7 of the *CSN2* gene was amplified using forward (5′-gat gaa ctc cag gat aaa atc – 3′) and reverse (5′-aat aat agg gaa ggg tcc ccg – 3′) primer designed with Primer3 software. The six sequence-specific hybridization fluorescence resonance energy transfer (FRET) probes were designed by TIB MOLBIOL GmbH (Berlin, Germany) using the genomic sequence from GenBank, Acc. No. M55158.1 (NCBI, USA). The hybridization probe system enabled distinguishing of three target SNP; g.8101C>A, (codon 67), g.8219C>A (codon 106), and g.8267C>G (codon 122) and to differentiate alleles *A*^1^, *A*^2^, *A*^3^, and *B*. Multiplex genotyping using 2 fluorescently labelled probes per each SNP was possible with a shorter sensor probe, that spans the polymorphic site and longer anchor probe. Continuous monitoring of the fluorescence during melting cycle demonstrated a sharp decrease in fluorescence when the detection probe dissociated from the template and FRET was interrupted. The real time PCR was performed in a 10-μL reaction mixture consisting of 20 to 30 ng genomic DNA, 0.3 U of *Taq* DNA polymerase, 30 μM of each dNTP, 0.2 μM of each primer and 0.2 μM of each probe.

### Milk and yoghurt analyses

The analyses of the good hygienic quality milk samples were performed at the National Reference Laboratory for Raw Milk (EN ISO 17025) of the Research Institute for Cattle Breeding in Rapotín, Czech Republic. The following methods used to determine the milk physiochemical and technological parameters were described in detail by Hanuš et al [14].

The active acidity (pH) was measured using a CyberScan 510 pH meter (Eutech Instruments, Thermo Fisher Scientific, Waltham, MA, USA). The TA was measured according to Soxhlet-Henkel with milk titration (100 mL) by an alkaline solution until the mixture was a light pink colour (in mL of the 0.25 mol/L NaOH/100 mL). The AS was expressed as the consumption of 96% ethanol used for MP precipitation for 5 mL of milk samples. The minerals (Ca, P, Na, Mg, and K) in mg/kg were quantified by atom absorption spectrophotometry with the Spectrometer SOLAAR S4 and 6F S97 Thermo Elemental (Eutech Instruments, Thermo Fisher Scientific, USA) according to the standard procedures in an accredited laboratory.

Yoghurt tests were carried out according to Czech milk industry standard ON 57 0534 [15]. Briefly, after heat treatment at 85°C for 5 min followed by cooling to 20°C, whole milk samples (50 mL) were inoculated with 2 mL of thermophilic lactic culture YC-180-40-FLEX (*Streptococcus thermophilus*, *Lactobacillus delbrűeckii subsp. lactis* and *L. d*. subsp. *bulgaricus*). Fermentation was performed at 43°C±2°C for 4 hours in a box air thermoregulator. The colony forming units (cfu) were calculated after 72 hours of classical plate cultivation (30°C) with GTK M agar according to Czech technical standard CSN ISO 6610 [16]. Then, the following yoghurt characteristics were measured: the active acidity (Y-pH), the titratable acidity according to Soxhlet-Henkel (Y-TA) and the total count of *Lactobacilli* (LC) and *Streptococci* (SC) strains in cfu/mL.

### Statistical analysis

Allele and genotype frequencies were obtained by allele counting at the *CSN3*, *CSN2*, and *LGB* loci. The χ^2^ test was carried out to determine whether the observed genotype frequencies deviated significantly from Hardy-Weinberg equilibrium. The association of the genetic polymorphism of the MPs with the investigated milk and yoghurt parameters was analysed using the general linear model (GLM) function in the statistical software programme SAS (Version 9.1, SAS Institute Inc., Cary, NC, USA). The model included the fixed effects of *CSN2*, *CSN3*, and *LGB* or the interactions among those three gene loci. The values of LC and SC were transformed to their decimal logarithm (log) to obtain normal value distribution.

The linear model used to investigate the relationship between genotypes and the observed parameters was as follows:

$${\text{y}}_{\text{ijk}}=\mu +{\text{HYS}}_{\text{i}}+{\text{G}}_{\text{j}}+{\text{bA}}_{\text{k}}+{\text{e}}_{\text{ijk}}$$

Where: y = observed milk or yoghurt parameter; μ = population mean; HYS = effect of herd, year and season of calving; G = genotype effect; bA = regression on age at first calving of cow; e = residual effect.

The effects of herds, year and season of calving were taken into account by creating herd–year–season classes (HYS). If included, the quality of the statistical model was improved because of high significance of the HYS effect. According to our previous findings and experience the proportion of variability of the tested traits explained by HYS effect is rather high (not published). The rare genotypes (frequency <1.3%) were not included in the statistical analysis. The statistical significance of the genotype effects of *CSN2*, *CSN3*, *LGB*, and the composite *CSN2-CSN3-LGB* was evaluated via an F-test.

## RESULTS AND DISCUSSION

### Descriptive statistics

Descriptive statistics for the milk physiochemical and fermentation ability parameters are presented in Table 1. Milk pH was found to be the most consistent trait among scientific reports, with ranges of values very similar to those in this study [7,17, 18]. The variation of milk TA was greater than for pH with 82.5% of samples surpassing the recommended range (6.2 to 7.8 °SH) [19]. According to de Marchi et al [20], a moderate increase in and imbalance of TA can be attributed to milk from first parturition cows. Additionally, Cassandro et al [21] described that variation of TA was greater than for pH in Holstein-Friesian cows. Tests of the AS serve as an important indicator of protein heat stability during milk processing [22], and they are widely adapted by the dairy industry [18]. The AS values were found to be lower than other results [23], indicating a small resistance of proteins to precipitation. Still Hanus et al [14] described the same average AS in the first calving cows of Czech Fleckvieh. Mean mineral concentrations of Ca, P, Na, Mg, and K in this study were comparable with the concentrations presented in other studies [24,25].

The acid fermentation test (i.e., yoghurt test) is generally applied to determine the usability of milk for fermented milk products. It is considered an important sign of quality that is taken into account in milk evaluation systems in the Czech Republic [26]. Yoghurt is a fermented gel made from milk using a starter culture mix of lactic acid bacteria, usually including *Lactobacillus bulgaricus* and *Streptococcus thermophilus*. The bacterial culture induces an acidification of the milk by the production of lactic acid from lactose, which leads to the aggregation of casein micelles into a 3-dimensional gel network [27]. Our measures of yoghurt acidity (Y-pH and Y-TA) and the quantities of the fermenting microorganisms of *Lactobacilli* and *Streptococci* were in accordance with Hanuš [14] and met the criteria for fresh yoghurt. The observed Y-pH values were slightly higher than those preferred by the current consumer, which are in the range of 4.2 to 4.4 [28]. This probably only reflects the fact that the cows in their first lactation were used in the present study, and later lactations may yield lower values.

### Genotype frequencies

The frequencies of the *CSN2*, *CSN3*, and *LGB* alleles and genotypes are presented in Table 2. The most frequent genotypes at the *CSN2* and *LGB* loci were both the heterozygotes *A*^1^*A*^2^ and *AB*, respectively. No individuals were found carrying the *A*^3^ allele. At the *CSN3* locus, *AA* and *AB* were the most common genotypes. The lowest occurrence among all the three genes investigated was recorded for the *CSN2 BB*, *CSN3 BE*, and *LGB AA* genotypes. The distribution of alleles and genotypes were almost the same as those previously described for Czech Fleckvieh cattle [4,12]. Similar results for the distribution of the *CSN2*, *CSN3*, and *LGB* genotypes were published in Simmental cows [2], which confirms a common genetic background for the two populations. Compared to other breeds, CFC showed a lower frequency of the *CSN3 E* allele, which had a negative effect on milk coagulation and processing properties [4]. The differences between the observed and the expected frequencies of the genes were not ascertained (p< 0.05), and the investigated population was in Hardy-Weinberg equilibrium. This result illustrates, that the evaluated generation of the CFC and genetic variation in the analyzed loci was not affected nor breeding for milk production nor other disturbing factors like are mutations, genetic drift and gene flow.

The allele frequencies affected the composite *CSN2-CSN3-LGB* genotypes. The resulting allelic combinations of the loci and their frequencies are reported in Table 3. A total of 49 different genotype combinations, including 24 rare ones, representing approximately 11% of the investigated cows, were identified. The most common combinations were those with *A*^2^*A*^2^ or *A*^1^*A*^2^ at *CSN2* and *AA* or *AB* at *CSN3* and *AB* at *LGB*; the genotypes *A*^1^*A*^2^ *AB* (9.6%), *A*^1^*A*^2^ *AA AB* and *A*^2^*A*^2^ *AB AB* (both 9.0%) were the most prevalent. In contrast, the genotypes with *A*^1^*B* or *A*^2^*B* at *CSN2* and *AE* or *BE* at *CSN3* were less frequent in the composite MP system. As in the study by Comin et al [6], the *BB* genotype of *CSN2* and the *EE* genotype of *CSN3* were very rare and never occurred together as a combination. Alleles *A* and *B* at the *LGB* locus were equally distributed across the existing genotype combinations. There is no report assigning the frequency of the *β*-CN – *κ*-CN – *β*-LG combinations in bovine milk. Nevertheless, nearly the same CN distribution pattern as the one we determined in CFC (frequency data are not shown) was published for Holsteins or Holstein crossbreds [3,5,6], suggesting a probable similar impact of breed-specific dairy selection programmes. However, our results differ from Gustavsson et al [17], who studied Finnish Ayrshire and Swedish Red cows and found a very high frequency of the *A*^1^*A*^2^ *AE* variant of the CN.

### Effects of a single gene on physiochemical and fermentative characteristics of milk

The results of the statistical analysis and the effects of *CSN2*, *CSN3*, and *LGB* on the investigated parameters, including F-test values, are presented in Table 4. Among all investigated physiochemical and fermentative characteristics, only the AS was significantly influenced by the *CSN3* genotype (p<0.05), while neither the *CSN2* nor *LGB* gene showed any significant effect. The stability of milk with the *CSN3 BE* genotype was the highest (0.51); conversely, the *BB* genotype showed the lowest stability (0.42). The most common genotype, *CSN3 AA*, achieved a middle value for AS (0.47). Botaro et al [18] tested ethanol stability in individual samples from Girolando and Holstein cows and found no effect of the *CSN3* genotype. Unlike in the present study, they have recorded decreasing milk stability according to genotype in the order *BB*>*AB*>*AA*. However, they did not analyse allele *E* of *CSN3*, so it was difficult to compare the results. The same authors [29] did not describe any relationship between the *LGB* genetic polymorphism and the milk AS, while our study has indicated a weak, non-significant relation (p>0.05) between the two parameters. Additionally, data from Imifidon et al [30] proved significant differences in stability among the different genetic types of *β*-LG. Cecchinato et al [31] investigated associations of allelic variants of the *LGB* with the titratable acidity in 294 Brown Swiss cows, which were sampled once. As in the present study, they reported no effect of *LGB* alleles on TA. Unfortunately, little knowledge exists about the influence of MP gene polymorphism on either AS or titratable acidity. Much attention has been paid to the other important variables influencing either the AS or the TA, such as milk yield [23], milk composition [22], breed, seasonality [18], and mineral content [32]. In accordance with van Hulzen et al [24], we propose that the observed impacts of the *CSN3* and *LGB* variants on the AS could be partly explained by the structural differences of the translated proteins, which can change their physiochemical properties, including stability.

Weak, non-significant relationships were observed between both *CSN3* variants and milk phosphorus (p>0.05) and between *LGB* variants and calcium content (p>0.05). The highest content of P was associated with *CSN3 BE*, while the lowest P was associated with *CSN3 AE*. The samples with *CSN3 BB* and *AB* genotypes contained comparable, intermediate P amounts. There was a likely favourable influence of the *CSN3 B* allele on the milk P content, while the negative effect of the *E* allele [4] was only partly confirmed. The Ca content of milk of different *LGB* genotypes was estimated as *BB*>*AB*>*AA*. Some authors have described significant associations between *β*-LG genetic variants and Ca and P contents, with the heterozygote *AB* being the superior genotype regarding their contents in milk [9]. On the other hand, Devod et al [8] did not observe any significant effect of either the *LGB* or the *CSN3* genotype on the Ca content. Our results seem to be close to those in the recently published review [33], which noted the direct effect of the major gene (genetic variants) on the minerals as well as the consequence for technological processing.

The parameters of milk FEA were not significantly affected by any of the genes. The statistical analysis showed only a trivial relationship between the *β*-CN genotype variants and the Y-pH (p>0.05) and the log LC (p>0.05) estimates. As shown in Table 4, the fermentation tests were performed correctly because they reflect an opposite relation between those two important technological characteristics.

### Effects of composite genotype on physiochemical and fermentative characteristics of milk

Altogether, 25 different *CSN2-CSN3-LGB* combinations with the frequency >1.3% were subjected to statistical analysis and gene effects estimation. The results of the statistical analysis and the obtained genotype effects including standard errors are summarized in Table 5. The observed effects of the composite genotypes mostly reflected the effects of the individual genes. Milk TA (p<0.01) was influenced the most, followed by Y-pH (p<0.05) and log LC (p<0.05). Additionally, a non-significant association (p>0.05) with P content was ascertained. However, contrary to the significant *CSN3* effect and the observed tendency for *LGB*, no relation between AS and the composite genotype was confirmed. The reason for this was unclear; we believe that the small size of the investigated population together with the exclusion of some rare but relevant genotypes could have influenced the AS variability and, consequently, the significance of the estimated genotype effect. As seen in Table 5, no association existed between the genotype and content of other minerals, pH, Y-TA, or log SC.

Although the present work involved a limited number of cows, the composite genotype effect on the TA was still demonstrated. In contrast to the action of the single genes, the interaction of the three genes was of considerable importance regarding this complex technological characteristic. The highest, and therefore in terms of milk quality the most favourable TA, was connected with the *A*^1^*A*^2^ *BB AA* genotype, while the lowest TA was found in the milk samples with the *A*^1^*A*^1^ *AE BB* genotype. Our findings are in contrast to those of Amigo et al [19], who found no significant association between *CSN2-CSN3-LGB* genotype and milk acidity in Fleckvieh cattle. On the other hand, Mayer et al [34] revealed that the interaction among the *CSN2*, *CSN3*, and *LGB* loci was highly correlated to the total protein, casein and whey protein contents of milk. Genčurová et al [26] emphasized that TA was positively correlated with protein and P content. Based on the results achieved, we could propose that genetic polymorphisms of the main MPs have an indirect effect on TA through control of the MP profile [17]. Except this, the milk acidity parameters such as pH and TA and the MP genetic polymorphism have been described as being strongly correlated to the coagulation traits of cheese-making [21,25]. De Marchi et al [20] reported that higher TA values were associated with better rennet-induced coagulation properties. According to our results, the MP genotype can be considered as another important factor influencing not only the TA values but also acid fermentation quality, which is important for yoghurt–making.

The P content has been shown to relate both to the *CSN3* genotype and to the composite genotype, although the genotype effects were non-significant in this study. According to Malcarne et al [32], the optimal milk is characterized by the highest contents of the major constituents, protein fractions and minerals, mainly P and Ca, and the highest values of TA. From this point of view, the best MP genotype combinations in this study were *A*^1^*A*^1^ *AA AB*, *A*^1^*A*^2^ *BB AA* and *A*^2^*A*^2^ *AB BB*. The worst values of both TA and P were conversely estimated for the *A*^1^*A*^1^ *AE BB*, *A*^2^*A*^2^ *AB AA* and *A*^2^*B AA AA* genotypes.

Yoghurt pH and log LC were the only fermentative characteristics related both to the *CSN2* and the composite genotype. Although the *CSN2* effects were not estimated as significant, the combination of the *CN* and *LGB* variants showed a strong influence on the fermentative ability with the yoghurt *Lactobacilli* culture (p<0.05). As is shown in Table 5, the statistical analysis noted the opposite relationship between estimates of the Y-pH and the log of the LC and a negative correlation between both parameters. The lowest, and thus better, Y-pH values and at the same time the highest count of fermenting LC were observed in the *A*^2^*B AA AA* and *A*^2^*B AA AB* genotypes; and vice versa for the *A*^1^*A*^2^ *BE BB* and *A*^1^*A*^1^ *AA AB* genotypes. However, those genotypes were shown to be rather infrequent in the investigated herds. The authors did not find relevant literature about the influence of genotype on both the yoghurt acidity and the number of fermenting microorganisms. Ketto et al [25] observed direct effects of *κ*-CN and the composite genotype of *α_S1_-β-κ*-CN on acid coagulation properties in Norwegian Red Cattle. According to Genčurova et al [26], the FEA parameters are considerably influenced in terms of casein content and fractions, the size of the casein micelles, the content of minerals and the yoghurt test itself. The growth and amount of lactic microflora depend especially on the hygienic quality of the milk and the content of undesirable components such as plant germicidal substances, feed preservatives, heavy metals and mycotoxins. Our findings indicated that the yoghurt fermentation test together with routine MP genotyping could contribute to milk quality control in the dairy industry. Moreover the detailed functional annotation of MP genes is important for genetic marker evaluation and dairy cattle selection [35].

## CONCLUSION

The present study was aimed at investigating the association between MP genotype and important milk physiochemical properties such as acidity, AS, and mineral content. Moreover, our results are the first to show a possible effect of *CN* and *LGB* gene polymorphisms on milk fermentation ability. The study confirmed that first parturition cows show a certain shift and imbalance in milk physiochemical parameters. The effect of the composite genotype on the investigated traits mostly reflected the effects of the individual genes. At the same time, the relationship between the physiochemical traits and FEA was apparent, and an interdependence of the fermentation parameters was confirmed. Genetic polymorphism has been related to the milk titratable acidity, AS, the contents of phosphorus and calcium in milk, yoghurt pH and the number of fermenting *Lactobacilli*. These findings support the previously accepted indirect effects of the MP allelic variants on the milk technological quality. Nevertheless, it could be advisable to perform a detailed analysis of polymorphisms and the interaction of the genes in larger populations to confirm the present initial results and to find differences among breeds. We suggest that further study of the MP composite genotypes in cattle may have future implications for the production of milk with defined characteristics.

## Figures and Tables

**Table 1:** Descriptive statistics for milk physiochemical parameters and fermentation ability (yoghurt test with microbial culture of *Lactobacilli* and *Streptococci*, n = 338)

Items	Mean	SD	Min	Max
Physiochemical parameters				
Active acidity (pH)	6.66	0.13	5.53	7.02
Titratable acidity1) (°SH, TA)	8.20	1.01	5.52	11.22
Alcohol stability2) (mL, AS)	0.43	0.18	0.16	1.02
Ca (mg/kg)	1,322.74	197.87	664.0	1,863.0
P (mg/kg)	1,121.62	166.59	740.0	1,675.0
Na (mg/kg)	367.29	78.39	228.0	782.0
Mg (mg/kg)	116.10	11.14	85.0	145.30
K (mg/kg)	1,617.95	178.94	1,017.0	1,989.0
Fermentation ability (yoghurt test)				
Active acidity (Y-pH)	4.87	0.18	4.46	5.30
Titratable acidity1) (°SH, Y-TA)	31.89	3.48	20.40	42.54
Total count of Lactobacilli (cfu/mL, LC)3)	2.75×10^7^	1.51×10^7^	4.5×10^6^	8.9×10^7^
Log LC4)	7.36	0.26	6.43	7.95
Total count of Streptococci (cfu/mL, SC)3)	6.60×10^8^	2.26×10^8^	2.0×10^8^	1.60×10^9^
Log SC4)	8.79	0.15	8.30	9.20

SD, standard deviation.

1) Consumption of 0.25 mol/L NaOH, which was used for the titration of 100 mL of milk or yoghurt (titratable acidity according to Soxhlet-Henkel).

2) Consumption of 96% ethanol used for protein coagulation in 5 mL of milk.

3) Colony forming units in 1 mL of yoghurt.

4) The values were transformed to the decimal logarithm scale.

**Table 2:** Observed and expected genotype and allele frequencies for *CSN2*, *CSN3*, and *LGB* loci (n = 338)

Locus	Genotype frequency1)			Allele frequency	
	
	Genotype	Observed	Expected	Allele	Frequency
*CSN2*	*A*^1^*A*^1^	0.118	0.118	*A*^1^	0.343
	*A*^1^*A*^2^	0.429	0.423	*A*^2^	0.617
	*A*^1^*B*	0.021	0.027	*B*	0.040
	*A*^2^*A*^2^	0.376	0.381		
	*A*^2^*B*	0.053	0.049		
	*BB*	0.003	0.002		
*CSN3*	*AA*	0.426	0.422	*A*	0.649
	*AB*	0.405	0.405	*B*	0.312
	*AE*	0.041	0.050	*E*	0.039
	*BB*	0.092	0.097		
	*BE*	0.036	0.024		
*LGB*	*AA*	0.254	0.237	*A*	0.487
	*AB*	0.465	0.500	*B*	0.513
	*BB*	0.281	0.264		

*CSN2*, β-casein; *CSN3*, *κ*-casein; *LGB*, *β*-lactoglobulin.

1) No observed genotype frequencies differed significantly from the expected frequencies (p<0.05, tested with a chi-squared test).

**Table 3:** Frequency distribution of the investigated milk protein genotype combinations of *CSN2 - CSN3 - LGB* (with frequency equal or higher then 0.013)

Genotype	Genotype frequency					

	*CSN3*					

	*AA*	*AB*	*AE*	*BB*	*BE*	
*CSN2*						*LGB*
*A*^1^*A*^1^	0.015	-	-	-	-	*AA*
*A*^1^*A*^2^	0.033	0.039	-	0.013	-	
*A*^2^*A*^2^	0.071	0.047	-	-	-	
*A*^2^*B*	0.013	-	-	-	-	
*A*^1^*A*^1^	0.015	0.027	-	-	-	*AB*
*A*^1^*A*^2^	0.083	0.089	-	0.024	0.013	
*A*^2^*A*^2^	0.060	0.083	-	-	-	
*A*^2^*B*	0.015	-	-	-	-	
*A*^1^*A*^1^	-	0.013	0.013	-	-	*BB*
*A*^1^*A*^2^	0.050	0.050	-	0.018	0.013	
*A*^2^*A*^2^	0.050	0.033	-	0.013	-	
Other1)	0.110	-	-	-	-	

*CSN2*, *β*-casein; *CSN3*, *κ*-casein; *LGB*, *β*-lactoglobulin.

1) Sum of genotype combinations with the very low frequency (<0.013). They were not included in the statistical analysis.

**Table 4:** The relation between genotype of *CSN2*, *CSN3*, and *LGB* and physiochemical and fermentation ability parameters1)

Genotype	Freq2)	Parameters3)											

		pH	TA	AS	Ca	P	Na	Mg	K	Y-pH	Y-TA	logLC	logSC
*CSN2*													
*A*^1^*A*^1^	0.12	6.63±0.02	8.00±0.18	0.45±0.02	1,346±33.6	1,136±27.1	366±12.9	119±1.98	1,632±31.6	4.89±0.03	30.7±0.55	7.28±0.04	8.78±0.03
*A*^1^*A*^2^	0.43	6.63±0.01	8.12±0.12	0.45±0.02	1,300±22.5	1,117±18.2	375±8.6	118±1.33	1,645±21.2	4.89±0.02	31.1±0.36	7.30±0.03	8.81±0.02
*A*^1^*B*	0.02	6.62±0.04	8.17±0.39	0.51±0.05	1,260±73.1	1,197±59.0	389±28.1	115±4.32	1,642±68.8	4.79±0.06	31.8±1.16	7.30±0.09	8.78±0.07
*A*^2^*A*^2^	0.38	6.62±0.01	7.90±0.13	0.44±0.02	1,308±23.8	1,108±19.2	382±9.2	117±1.41	1,618±22.4	4.88±0.02	31.6±0.37	7.32±0.03	8.81±0.02
*A*^2^*B*	0.05	6.66±0.03	7.92±0.25	0.52±0.03	1,310±47.1	1,134±38.0	353±18.1	112±2.78	1,581±44.3	4.81±0.04	32.9±0.74	7.46±0.06	8.87±0.04
F test		0.41	1.29	1.18	0.91	0.82	0.93	1.44	0.80	1.88 !	1.74	2.10 !	0.69
*CSN3*													
*AA*	0.43	6.63±0.01	8.02±0.11	0.47±0.02	1,287±22.5	1,122±18.0	379±8.6	117±1.33	1,625±21.2	4.89±0.02	31.3±0.35	7.32±0.03	8.82±0.02
*AB*	0.41	6.63±0.01	8.05±0.12	0.43±0.02	1,325±23.5	1,139±18.8	375±9.0	119±1.38	1,635±22.2	4.87±0.02	31.6±0.37	7.32±0.03	8.81±0.02
*AE*	0.04	6.64±0.03	7.61±0.29	0.43±0.04	1,318±51.8	1,017±41.5	369±19.9	112±3.06	1,634±48.9	4.88±0.05	30.6±0.86	7.32±0.06	8.77±0.05
*BB*	0.09	6.61±0.02	8.11±0.21	0.42±0.03	1,298±38.5	1,136±30.8	387±14.8	118±2.28	1,648±36.4	4.85±0.03	31.1±0.61	7.30±0.05	8.77±0.04
*BE*	0.04	6.64±0.03	7.78±0.31	0.51±0.04	1,325±56.3	1,145±45.0	351±21.6	119±3.32	1,621±53.2	4.95±0.05	30.7±0.89	7.20±0.06	8.73±0.05
*F test*		0.25	0.79	2.76 *	0.76	2.11 !	0.60	1.32	0.14	1.08	0.59	0.89	1.36
*LGB*													
*AA*	0.25	6.64±0.02	7.90±0.14	0.47±0.02	1,281±26.1	1,105±21.2	376±10.0	116±24.7	1,629±24.7	4.87±0.02	31.5±0.43	7.35±0.03	8.82±0.03
*AB*	0.46	6.63±0.01	8.09±0.11	0.46±0.02	1,293±22.4	1,125±18.2	383±8.6	116±21.2	1,625±21.2	4.88±0.02	31.4±0.35	7.32±0.02	8.81±0.02
*BB*	0.28	6.62±0.01	7.97±0.13	0.43±0.02	1,339±24.4	1,123±19.8	367±9.4	119±23.1	1,638±23.1	4.89±0.02	31.3±0.38	7.29±0.03	8.80±0.02
*F test*		0.82	1.06	2.42 !	2.51 !	0.51	1.40	2.05	0.14	0.11	0.11	1.83	0.35

*CSN2*, *β*-casein; *CSN3*, *κ*-casein; *LGB*, *β*-lactoglobulin; TA, titratable acidity; AS, alcohol stability; Y-pH, yoghurt pH; Y-TA, yoghurt titratable acidity; log LC, log of total count of *Lactobacilli* (cfu/mL); log SC, log of total count of *Streptococci* (cfu/mL).

1) For explanation of the physiochemical and fermentation parameters see Table 1.

2) Frequency of the genotype.

3) Values are least squares means±standard error.

! 0.10≥p>0.05.

* 0.05≥p>0.01.

** 0.01≥p>0.001.

**Table 5:** The relation between composite *CSN2 – CSN3 – LGB* genotype and physiochemical and fermentation ability parameters1)

Genotype	Freq2)	Parameters3)											

		pH	TA	AS	Ca	P	Na	Mg	K	Y-pH	Y-TA	logLC	logSC
*A*^1^*A*^1^ *AA AA*	0.016	6.68±0.05	7.42±0.49	0.56±0.06	1,244±88.4	1,141±67.4	400±32.5	124±5.0	1,620±81.5	4.80±0.08	30.6±1.50	7.42±0.11	8.90±0.09
*A*^1^*A*^1^ *AA AB*	0.016	6.63±0.05	8.48±0.49	0.44±0.06	1,335±87.8	1,195±66.9	369±32.3	118±5.0	1,612±80.9	5.00±0.08	28.5±1.49	7.23±0.11	8.70±0.09
*A*^1^*A*^1^ *AB AB*	0.029	6.68±0.04	7.91±0.33	0.43±0.05	1,307±66.9	1,108±51.0	354±24.6	115±3.8	1,653±61.7	4.88±0.06	30.4±1.07	7.30±0.08	8.76±0.07
*A*^1^*A*^1^ *AB BB*	0.013	6.59±0.06	7.82±0.50	0.45±0.07	1,379±99.9	1,154±76.2	365±36.8	131±5.6	1,667±92.2	4.77±0.08	32.7±1.52	7.47±0.11	8.81±0.10
*A*^1^*A*^1^ *AE BB*	0.013	6.66±0.07	6.33±0.56	0.38±0.08	1,520±111.6	999±85.1	338±41.1	106±6.3	1,524±103	4.81±0.09	30.7±1.70	7.36±0.12	8.83±0.11
*A*^1^*A*^2^ *AA AA*	0.035	6.69±0.04	8.16±0.30	0.61±0.04	1,251±60.7	1,110±46.3	363±22.3	116±3.4	1,654±56.0	4.94±0.05	30.7±0.93	7.48±0.07	8.88±0.06
*A*^1^*A*^2^ *AA AB*	0.090	6.67±0.02	7.97±0.20	0.49±0.03	1,263±40.7	1,083±31.0	414±15.0	114±2.3	1,626±37.5	4.92±0.03	30.3±0.62	7.35±0.04	8.89±0.04
*A*^1^*A*^2^ *AA BB*	0.054	6.62±0.03	8.16±0.25	0.47±0.03	1,326±48.9	1,121±37.3	368±18.0	119±2.8	1,653±45.1	4.87±0.04	31.8±0.75	7.27±0.05	8.78±0.05
*A*^1^*A*^2^ *AB AA*	0.042	6.63±0.03	7.49±0.30	0.48±0.04	1,266±57.9	1,046±44.1	395±21.3	117±3.3	1,529±53.4	4.81±0.05	31.1±0.92	7.35±0.07	8.80±0.06
*A*^1^*A*^2^ *AB AB*	0.096	6.64±0.02	8.10±0.20	0.46±0.03	1,342±38.6	1,146±29.4	368±14.2	121±2.2	1,656±35.6	4.94±0.03	30.4±0.62	7.28±0.04	8.81±0.04
*A*^1^*A*^2^ *AB BB*	0.054	6.62±0.03	8.23±0.25	0.40±0.04	1,385±49.8	1,169±38.0	346±18.3	118±2.8	1,662±46.0	4.87±0.04	31.1±0.76	7.28±0.05	8.80±0.05
*A*^1^*A*^2^ *BB AA*	0.013	6.59±0.07	9.04±0.56	0.42±0.08	1,381±111.7	1,172±85.1	422±41.1	126±6.3	1,757±103	4.87±0.09	31.5±1.71	7.24±0.12	8.73±0.11
*A*^1^*A*^2^ *BB AB*	0.026	6.61±0.04	8.06±0.36	0.45±0.05	1,172±72.0	1,109±54.9	340±26.5	113±4.1	1,742±66.4	4.87±0.06	29.9±1.10	7.32±0.08	8.79±0.07
*A*^1^*A*^2^ *BB BB*	0.019	6.60±0.05	7.75±0.40	0.43±0.06	1,315±79.9	1,037±60.9	435±29.4	116±4.5	1,645±73.7	4.80±0.06	30.6±1.22	7.33±0.09	8.77±0.08
*A*^1^*A*^2^ *BE AB*	0.013	6.61±0.06	7.45±0.50	0.56±0.07	1,331±99.0	1,042±75.4	378±36.4	117±5.6	1,602±91.2	4.79±0.08	30.2±1.53	7.24±0.11	8.68±0.10
*A*^1^*A*^2^ *BE BB*	0.013	6.69±0.07	7.98±0.56	0.47±0.08	1,266±112.4	1,290±85.7	318±41.4	120±6.3	1,723±104	5.14±0.09	29.7±1.72	7.09±0.12	8.72±0.11
*A*^2^*A*^2^ *AA AA*	0.077	6.66±0.03	7.94±0.22	0.48±0.03	1,286±42.7	1,117±32.5	360±15.7	116±2.4	1,647±39.3	4.95±0.04	30.2±0.66	7.28±0.05	8.80±0.04
*A*^2^*A*^2^ *AA AB*	0.067	6.60±0.03	8.10±0.24	0.49±0.03	1,273±47.0	1,103±35.8	383±17.3	118±2.6	1,615±43.3	4.87±0.04	31.9±0.72	7.27±0.05	8.78±0.05
*A*^2^*A*^2^ *AA BB*	0.054	6.61±0.03	7.21±0.25	0.45±0.04	1,368±50.0	1,025±37.8	384±18.3	118±2.8	1,637±45.8	4.89±0.04	30.7±0.76	7.42±0.05	8.81±0.05
*A*^2^*A*^2^ *AB AA*	0.051	6.63±0.03	7.12±0.26	0.45±0.04	1,326±51.8	1,058±39.5	404±19.1	113±2.9	1,646±47.8	4.83±0.04	31.6±0.79	7.40±0.06	8.82±0.05
*A*^2^*A*^2^ *AB AB*	0.090	6.65±0.02	7.91±0.21	0.45±0.03	1,301±41.0	1,135±31.3	407±15.1	117±2.3	1,615±37.9	4.83±0.03	32.2±0.63	7.42±0.05	8.85±0.04
*A*^2^*A*^2^ *AB BB*	0.035	6.64±0.04	8.42±0.32	0.46±0.05	1,324±63.1	1,143±48.1	366±23.2	123±3.6	1,623±58.2	4.98±0.05	29.1±0.97	7.31±0.70	8.80±0.06
*A*^2^*A*^2^ *BB BB*	0.013	6.68±0.06	7.24±0.48	0.43±0.07	1,319±96.4	1,057±73.5	388±35.5	108±5.4	1,489±89.0	4.79±0.08	31.2±1.47	7.34±0.11	8.78±0.09
*A*^2^*B AA AA*	0.013	6.68±0.07	7.02±0.57	0.52±0.08	1,326.6±113.5	1,069±86.6	358±41.8	114±6.4	1,457±105	4.76±0.10	32.8±1.73	7.64±0.12	8.93±0.11
*A*^2^*B AA AB*	0.016	6.67±0.05	7.30±0.44	0.58±0.06	1,369±87.8	1,141±66.0	368±32.3	107±5.0	1,442±81.0	4.75±0.07	32.6±1.35	7.57±0.10	8.85±0.08
F test		0.71	1.84 **	1.21	0.73	1.44 !	1.30	1.28	1.00	1.52 *	0.95	1.50 *	0.64

*CSN2*, *β*-casein; *CSN3*, κ-casein; *LGB*, β-lactoglobulin; TA, titratable acidity; AS, alcohol stability; Y-pH, yoghurt pH; Y-TA, yoghurt titratable acidity; log LC, log of total count of *Lactobacilli* (cfu/mL); log SC, log of total count of *Streptococci* (cfu/mL).

1) Values are least squeares means±standard error.

2) Frequency of the genotype.

3) For explanation of the physiochemical and fermentative parameters see Table 1.

! 0.10≥p>0.05.

* 0.05≥p>0.01.

** 0.01≥p>0.001.
